# Effects of Supervised Exercise Therapy on Muscle Function During Walking in Patients with Peripheral Artery Disease

**DOI:** 10.3390/bioengineering11111103

**Published:** 2024-10-31

**Authors:** Cody P. Anderson, Iraklis I. Pipinos, Jason M. Johanning, Sara A. Myers, Hafizur Rahman

**Affiliations:** 1 School of Health and Kinesiology, University of Nebraska at Omaha, Omaha, NE 68182, USA; codypanderson@unomaha.edu; 2 Department of Surgery, University of Nebraska Medical Center, Omaha, NE 68198, USA; ipipinos@unmc.edu (I.I.P.); jjohanning@unmc.edu (J.M.J.); 3 Department of Surgery and Research Service, Nebraska-Western Iowa Veterans Affairs Medical Center, Omaha, NE 68105, USA; samyers@unomaha.edu; 4 Department of Biomechanics, University of Nebraska at Omaha, Omaha, NE 68182, USA; 5 School of Podiatric Medicine, University of Texas Rio Grande Valley, Harlingen, TX 78550, USA

**Keywords:** peripheral artery disease, supervised exercise therapy, gait biomechanics, musculoskeletal modeling and simulation, muscle function

## Abstract

Background: Although supervised exercise therapy (SET) is a primary treatment for peripheral artery disease (PAD), the current literature is limited regarding the mechanisms contributing to increased walking distances, including how lower extremity muscle function is altered after SET. This study aimed to investigate the effects of SET on lower extremity muscle function during walking in patients with PAD. Methods: Twelve patients with PAD participated in a 6-month SET program consisting of three weekly exercise sessions (a total of 72 sessions) and adhered to the American College of Sports Medicine’s (ACSM) recommendations. Each session started with a 5 min warm-up of mild walking and static stretching of upper and lower body muscles, followed by 50 min of intermitted exercise on a treadmill, and then finished with 5 min of cool-down activities similar to the warm-up. Each patient walked across a 10 m pathway with reflective markers on their lower limbs twice: before (baseline) and after six months of participation in SET (post-exercise). Marker coordinates and ground reaction forces were recorded and imported to OpenSim software (version 4.0) for gait simulations. Muscle force, muscle power, and metabolic rate were estimated from OpenSim and compared between the baseline and post-exercise. Results: The mean plantar flexor force was not altered after SET. However, individuals’ plantar flexor muscles demonstrated improvements in force production (lateral gastrocnemius: 75–80% of stance, Cohen’s d = 0.20–0.43; medial gastrocnemius: 65–85% of stance, Cohen’s d = 0.20–0.71; soleus: 90–95% of stance, Cohen’s d = 0.20–0.26). Furthermore, plantar flexor power increased (80–95% of stance, Cohen’s d = 0.20–0.39) and this was attributed to increased power in the lateral gastrocnemius (80–85% of stance, Cohen’s d = 0.20–0.47), medial gastrocnemius (80–90% of stance, Cohen’s d = 0.22–0.60), and soleus muscles (85–95% of stance, Cohen’s d = 0.20–0.49). Similarly, other muscle groups (knee extensors, knee flexors, hip abductors, hip adductors, hip extensors, and hip flexors) also exhibited force and power increases after SET. Additionally, force and power variances were significantly decreased in several muscle groups (plantar flexors, knee extensors, hip abductors, hip external rotators, hip extensors, and hip flexors). Total metabolic rate also increased during the stance period where muscle force and power were elevated after SET (early stance: 5–25%, Cohen’s d = 0.20–0.82; mid stance: 35–45%, Cohen’s d = 0.20–0.47; late stance: 75–80%, Cohen’s d = 0.20–0.36). Conclusions: Our results suggest that from a biomechanics perspective, muscle functions during walking are improved in patients with PAD after SET; however, the improvements were generally small and were not reflected by all muscle groups.

## 1. Introduction

Peripheral artery disease (PAD) is a common cardiovascular disorder that affects approximately 8 million people in the United States and more than 200 million individuals worldwide [1,2,3]. PAD manifests from the development of atherosclerotic plaques in conduit arteries, which reduces blood flow to the legs, and commonly results in ischemic muscle pain known as intermittent claudication. Intermittent claudication, an intense cramping-like pain in the muscles, typically arises during physical exertion and limits exercise capacity. Furthermore, patients with PAD have an altered walking performance, decreased muscle strength, reduced quality of life, a progressively sedentary lifestyle, and a higher risk of mortality compared to individuals of similar ages without PAD [4,5,6,7,8,9,10,11,12,13,14]. Patients with PAD walk slowly with impaired gait biomechanics, including altered joint angles, reduced joint torque, and reduced joint power at the ankle, knee, and hip joints [4,7,12,13,14]. The plantar flexor muscle strength is reduced in patients with PAD with a significant decrease in propulsion force during push-off compared to healthy individuals [10,15].

Supervised exercise therapy (SET) is a primary treatment for patients with PAD [16,17,18,19]. Previous studies from our group and others have reported significant improvements in quality of life and walking distance in patients with PAD after SET [20,21,22,23,24,25]. We found that walking speed significantly improved, by 4.5%, following a 6-month SET program [12]. Advanced biomechanical analysis demonstrated that gait biomechanics significantly improved after SET [20]. Additionally, isometric strength testing shows that SET strengthens ankle plantar flexor muscles and augments maximal force [20]. The improvements in muscle strength may be the primary contributor to an improved walking performance.

The current literature is limited in terms of explaining mechanisms contributing to increased walking performance, including how lower extremity muscle functions are altered during walking in patients with PAD after SET. Our previous results suggest that patients with PAD generate inadequate force and power with the muscles that surround the ankle, knee, and hip joints compared to age-matched healthy older individuals [26]. However, it is not clear how the muscle forces and powers change during walking after SET. Musculoskeletal modeling and simulation have the potential to fill this gap by providing important information about the individual and combined muscle forces and powers during walking. A better understanding of changes in muscle function during walking after SET may provide pertinent insight into the mechanisms of gross biomechanical improvement after SET and can further play an important role in developing interventions for patients with PAD. Therefore, the aim of this study was to use musculoskeletal modeling and simulation to investigate the effects of SET on lower extremity muscle functions during walking in patients with PAD. We hypothesized that muscle functions would improve following SET.

## 2. Materials and Methods

### 2.1. Participants and SET Protocol

This study was approved by the Institutional Review Boards at the Nebraska-Western Iowa Veterans Affairs Medical Center and the University of Nebraska Medical Center. A total of 12 patients (age: 67.08 ± 5.85 years, body mass: 93.55 ± 18.19 kg, body mass index: 30.60 ± 6 kg/m^2^), diagnosed with Fontaine stage II PAD, were recruited through the vascular surgery clinics at the two study sites. Recruited patients had not previously participated in any SET programs or revascularization treatments prior to enrollment in this study. Patients’ consent was obtained before study participation. Patients’ history and physical examination were evaluated by one of two board-certified vascular surgeons.

The detailed SET protocol utilized in this study has previously been described [20]. Each patient participated in a six-month SET program that consisted of three exercise sessions per week (a total of 72 sessions) and adhered to the American College of Sports Medicine’s (ACSM) recommendations, which aligns with previous studies that demonstrated improvements in walking distances [27,28]. Each exercise session started with a 5 min warm-up of mild walking and static stretching of upper and lower body muscles. Then, patients participated in 50 min of intermittent exercise on a treadmill. At the beginning of the exercise, the treadmill speed was set to 2 mph or lower if desired by the patient at 0% grade. Patients walked until claudication pain reached a moderate to severe level, took rest until claudication pain was relieved, and then continued walking again for up to 50 min with rest as needed. The SET program progressed after a patient walked for 8 to 10 continuous minutes at the initial workload (2 mph at 0% grade) by increasing the grade by 1% to 2% or the speed by 0.5 mph as tolerated by the patient. The exercise session ended with 5 min cool-down activities similar to the warm-up.

### 2.2. Experimental Data Collection

Experimental tests were conducted at the Biomechanics Research Building at the University of Nebraska at Omaha. Each patient was evaluated twice: before (baseline) and after six months of SET (post-exercise). Upon arrival at the laboratory, subjects’ height, body mass, and anthropometric measurements were recorded. Reflective markers were placed on specific anatomical locations of each subject’s lower limbs as previously described [4,7]. Patients walked across a 10 m pathway with force plates mounted level with the ground. It was ensured that we obtained at least one clean foot contact for the affected leg and most affected leg for patients with unilateral PAD and patients with bilateral PAD, respectively. A clean foot contact was defined when one leg completely landed on a single force plate during the stance phase with no other contact on that force plate during the walking trial. The coordinates of the markers were recorded using a 12 high-speed infrared camera system (60 Hz, Motion Analysis Corporation, Rohnert Park, CA, USA). The ground reaction forces were recorded by force plates (600 Hz, AMTI, Watertown, MA, USA).

### 2.3. Musculoskeletal Modeling and Simulation

Gait simulations were performed in OpenSim version 4.0, an open-source musculoskeletal modeling and simulation platform [29]. A generic ‘Full Body Musculoskeletal Model’ developed by Rajagopal et al. was used to perform walking simulations [30]. This model is comprised of 80 muscle–tendon units with 23 degrees of freedom to define the joint movements.

First, the anthropometry of the generic model was scaled to the anthropometry of each individual subject by using the ‘Scale’ tool in OpenSim. The dimensions of each model segment were scaled based on the distances between marker pairs obtained from the static experimental trial, which corresponded to virtual marker locations on the OpenSim model. Experimental marker data were imported into the ‘Inverse Kinematics’ tool to calculate the joint angles by minimizing errors between the experimental marker trajectories and virtual markers on the scaled model. Joint angles obtained from the inverse kinematics results for walking trials were imported into the ‘Residual Reduction Algorithm’ tool in OpenSim. The residual reduction algorithm minimizes the effects of modeling and marker data processing errors that aggregate and generate nonphysical compensatory forces called residuals. The kinematics obtained from the residual reduction algorithm were imported into the ‘Computed Muscle Control (CMC)’ tool to calculate the individual muscle forces. Muscle force is defined as the force exerted by the corresponding muscle during the stance phase of walking, and muscle power is calculated as the product of force and velocity. The metabolic probe was added to the muscles in the model to estimate the energy consumption of each muscle [31,32,33]. This metabolic probe estimates the rate of muscle energy expenditure for each muscle by considering activation heat rate, maintenance heat rate, shortening/lengthening heat rate, and mechanical work rate of the contractile elements in the muscle model [31].

### 2.4. Data Processing

Parameters (force, power, and metabolic rate) for the lower extremity muscles were exported by the CMC tool, and the output files were further processed by custom routines in MATLAB (R2022a, The MathWorks, Inc., Natick, MA, USA) and Python (version 3.1, Python Software Foundation, Wilmington, DE, USA). Force and power time series data were sorted and compiled for the following muscle groups: ankle plantar flexors, knee extensors, knee flexors, hip abductors, hip adductors, hip external rotators, hip extensors, and hip flexors. The muscle groups were based on pre-defined muscle groups in the OpenSim model. Only for the ankle plantar flexor muscle group, individual muscle contributions (force and power) for lateral gastrocnemius, medial gastrocnemius, and soleus were exported. Out of twelve patients, five patients had unilateral PAD, and from those, only the affected legs were included for analysis. The remaining seven patients had bilateral PAD; we analyzed only the most affected leg from each patient based on the ankle-brachial index.

The time series data obtained from the CMC tool were resampled to 1000 data points and expressed as a percentage of stance, which is defined as the portion of gait between heel-strike and toe-off of the analyzed leg. Resampling was performed to ensure that each relative time point could be compared between subjects and groups. Muscle forces and powers were normalized by the subjects’ bodyweights in newtons and kilograms, respectively. The total metabolic rate was exported from OpenSim as a time series representing the arithmetic sum of the metabolic rates of all the individual muscles in the model during the stance phase.

### 2.5. Statistical Analysis

Cohen’s d effect size was calculated at each time point during the stance phase between the average baseline and post-exercise values for muscle force, muscle power, and metabolic rate. Cohen’s d effect sizes between 0.20 and 0.50, 0.50 and 0.80, and greater than 0.80 were classified as small, medium, and large effects, respectively [34]. Effect sizes were used as an indication of group mean changes after SET. The range of Cohen’s d values across the stance phase were reported.

Additionally, F-tests (ratio of the variances between the baseline and the post-exercise groups) were performed at each time point during the stance phase in the time series data to determine changes from the baseline to post-exercise in the variance of muscle force, muscle power, and metabolic rate. Since walking performance can be highly variable among patients with PAD, a change in variance after SET may indicate an effect of the intervention. Significance for the F-test was set at *p* < 0.05. Normality was assessed with the Shapiro–Wilk test. All statistical tests were performed in GraphPad Prism software (version 9.0.2 for Windows, GraphPad Software, San Diego, CA, USA).

## 3. Results

### 3.1. Ankle Plantar Flexors

**Figure 1:**
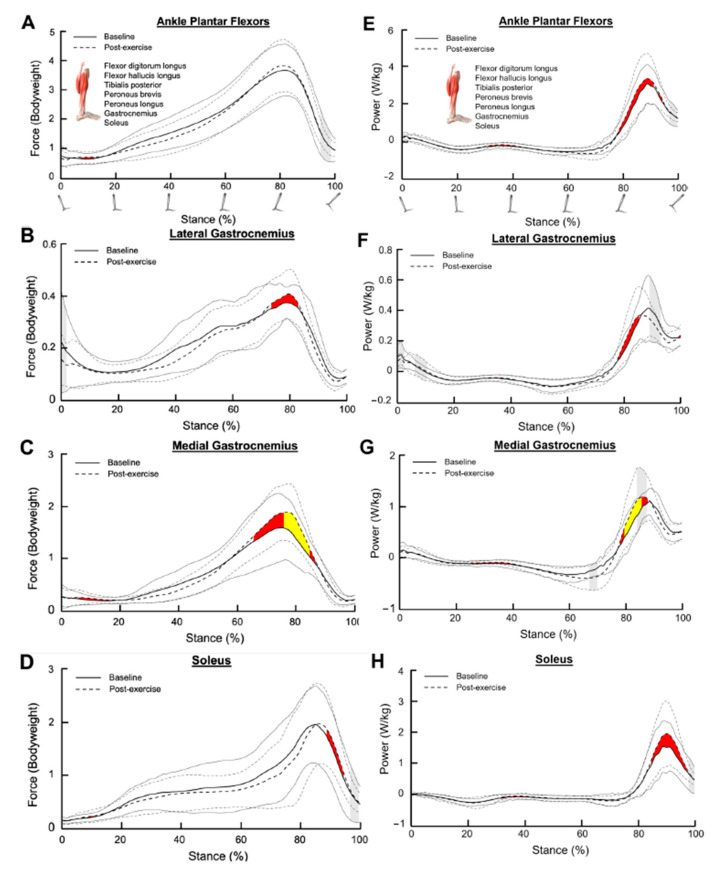
Muscle forces for (**A**) combined ankle plantar flexors, (**B**) lateral gastrocnemius, (**C**) medial gastrocnemius, and (**D**) soleus during the stance phase. (**E**–**H**) represent the muscle powers for the combined ankle plantar flexors, lateral gastrocnemius, medial gastrocnemius, and soleus, respectively, during the stance phase. The dark solid line and the dark dotted line represent the average muscle force/power at the baseline and post-exercise sessions, respectively. The grey solid lines and the grey dotted lines represent one standard deviation from the average values. Shaded red and shaded yellow regions indicate small and medium size effects, respectively, determined by Cohen’s d values. Shaded grey regions between standard deviation lines indicate a significant difference in muscle force/power variance between the baseline and post-exercise sessions (*p* < 0.05).

Total ankle plantar flexor force during late stance did not have an effect size greater than 0.20 (Figure 1A). However, Cohen’s d revealed small and medium effect sizes for the changes in lateral and medial gastrocnemius and soleus forces during late stance (lateral gastrocnemius: 75–80% of stance, Cohen’s d = 0.20–0.43; medial gastrocnemius: 65–85% of stance, Cohen’s d = 0.20–0.71; soleus: 90–95% of stance, Cohen’s d = 0.20–0.26; Figure 1B–D).

There was a small effect size for the changes in peak ankle plantar flexor power in late stance (80–95% of stance, Cohen’s d = 0.20–0.39, Figure 1E). The lateral gastrocnemius exhibited a small effect size for changes in power generation during late stance (80–85% of stance, Cohen’s d = 0.20–0.47, Figure 1F). The medial gastrocnemius exhibited small to medium size effects for changes in power generation during late stance (80–90% of stance, Cohen’s d = 0.22–0.60, Figure 1G). Finally, the soleus exhibited small effect sizes for changes in power generation during late stance (85–95% of stance, Cohen’s d = 0.20–0.49, Figure 1H).

Small regions of stance demonstrated significant changes in variance (*p* < 0.05), with the medial gastrocnemius having the most meaningful changes in power variance. Variance was significantly elevated during power absorption (70% of stance) and production (85% of stance) after SET.

### 3.2. Knee Extensors

**Figure 2:**
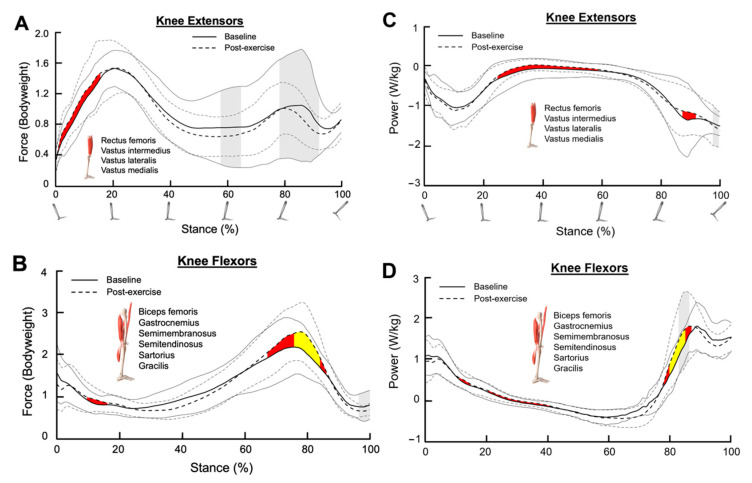
Muscle forces for (**A**) combined knee extensors and (**B**) combined knee flexors during the stance phase. (**C**,**D**) represent the muscle powers for the combined knee extensors and the combined knee flexors, respectively, during the stance phase. The dark solid line and the dark dotted line represent the average muscle force/power at the baseline and post-exercise sessions, respectively. The grey solid lines and the grey dotted lines represent one standard deviation from the average values. Shaded red and shaded yellow regions indicate small and medium size effects, respectively, determined by Cohen’s d values. Shaded grey regions between standard deviation lines indicate a significant difference in muscle force/power variance between the baseline and post-exercise sessions (*p* < 0.05).

Cohen’s d revealed a small effect size for the changes in knee extensor force during weight acceptance (0–15% of stance, Cohen’s d = 0.21–0.47, Figure 2A). Knee extensor power also exhibited a small effect size for the increase in power production during mid-stance after SET (25–60% of stance, Cohen’s d = 0.20–0.46, Figure 2C).

Although knee extensor power variance was not changed after SET (Figure 2C), significant reductions in knee extensors force variance in regions between 60 and 90% of stance were observed after SET (*p* < 0.05, Figure 2A).

### 3.3. Knee Flexors

Cohen’s d revealed small to medium effect sizes for the increase in knee flexor force during late stance (65–85% of stance, Cohen’s d = 0.26–0.68, Figure 2B). Similarly, knee flexor power was also elevated during the late stance after SET (80–90% of stance, Cohen’s d = 0.21–0.57, Figure 2D).

Knee flexor force variance remained largely unaltered after SET; however, knee flexor power variance was significantly increased after SET at 85% of stance (*p* < 0.05, Figure 2B,D).

### 3.4. Hip Abductors

**Figure 3:**
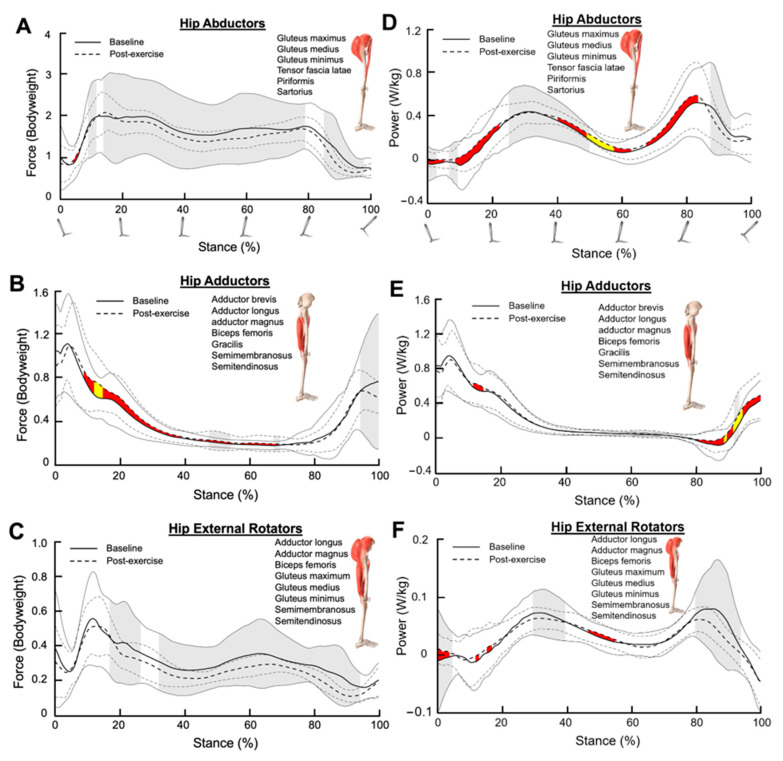
Muscle forces for (**A**) combined hip abductors, (**B**) combined hip adductors, and (**C**) combined hip external rotators during the stance phase. (**D**–**F**) represent the muscle powers for the combined hip abductors, the combined hip adductors, and the combined hip external rotators, respectively, during the stance phase. The dark solid line and the dark dotted line represent the average muscle force/power at the baseline and post-exercise sessions, respectively. The grey solid lines and the grey dotted lines represent one standard deviation from the average values. Shaded red and shaded yellow regions indicate small and medium size effects, respectively, determined by Cohen’s d values. Shaded grey regions between standard deviation lines indicate a significant difference in muscle force/power variance between the baseline and post-exercise sessions (*p* < 0.05).

There were no changes in mean hip abductor force after SET according to Cohen’s d (Figure 3A). In contrast, there were small to medium effect sizes corresponding to increased hip abductor power throughout the stance phase after SET (early stance: 0–25%, Cohen’s d = 0.20–0.48; mid stance: 40–60%, Cohen’s d = 0.20–0.72; late stance: 70–85%, Cohen’s d = 0.20–0.41; Figure 3D).

Hip abductor force variance decreased significantly after SET during the majority of the stance phase (10–95% of stance, *p* < 0.05, Figure 3A). Similarly, hip abductor power variance decreased significantly after SET during the mid-stance phase (30–50% of stance, *p* < 0.05, Figure 3D).

### 3.5. Hip Adductors

Hip adductor force increased during the early and mid-stance phases after SET, with small to medium effect sizes (10–70% of stance, Cohen’s d = 0.20–0.58, Figure 3B). Hip adductor power also increased during the late stance phase after SET, where effect sizes were small to medium (80–100% of stance, Cohen’s d = 0.20–0.57, Figure 3E). Hip adductor force and power variance remained largely unaltered after SET.

### 3.6. Hip External Rotators

Mean hip external rotator force was unaltered after SET (Figure 3C). Hip external rotator power was also unaltered in a meaningful way after SET (Figure 3F).

Hip external rotator force variance was significantly decreased after SET from 15 to 95% of the stance phase (*p* < 0.05, Figure 3C). Hip external rotator power variance was also significantly decreased after SET from 30 to 40% and from 80 to 95% of the stance phase (*p* < 0.05, Figure 3F).

### 3.7. Hip Extensors

**Figure 4:**
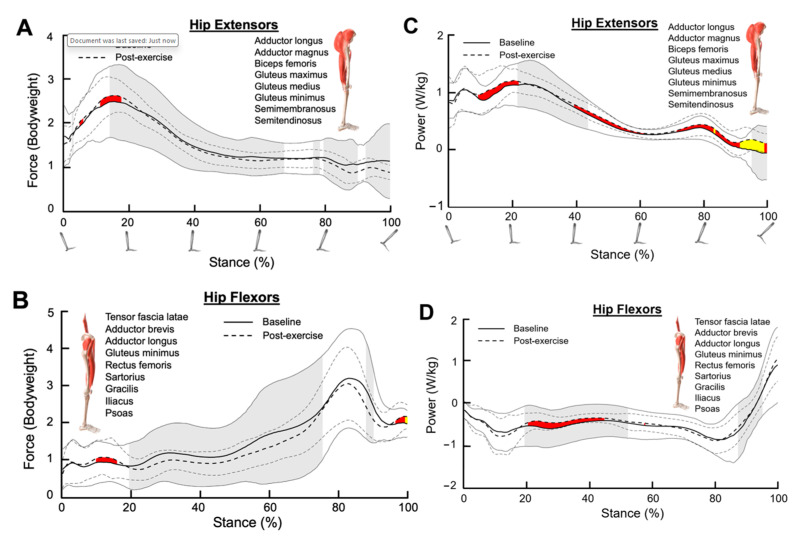
Muscle forces for (**A**) combined hip extensors and (**B**) combined hip flexors during the stance phase. (**C**,**D**) represent the muscle powers for the combined hip extensors and the combined hip flexors, respectively, during the stance phase. The dark solid line and the dark dotted line represent the average muscle force/power for the baseline and post-exercise sessions, respectively. The grey solid lines and the grey dotted lines represent one standard deviation from the average values. Shaded red and shaded yellow regions indicate small and medium size effects, respectively, determined by Cohen’s d values. Shaded grey regions between standard deviation lines indicate a significant difference in muscle force/power variance between the baseline and post-exercise sessions (*p* < 0.05).

Hip extensor force increased only during the early stance phase after SET, consistent with a small effect size according to Cohen’s d (15–20% of stance, Cohen’s d = 0.20–0.23, Figure 4A). However, hip extensor power increased throughout the stance phase after SET, consistent with small to medium effect sizes (early stance: 10–20%, Cohen’s d = 0.20–0.49; mid stance: 40–60%, Cohen’s d = 0.20–0.45; late stance: 70–100%, Cohen’s d = 0.20–0.71; Figure 4C).

Both hip extensor force variance and hip extensor power variance decreased significantly after SET (force: 15–100% of stance; power: 20–40% of stance, *p* < 0.05; Figure 4A,C).

### 3.8. Hip Flexors

Mean hip flexor force was not meaningfully altered after SET (Figure 4B). However, hip flexor power was increased during the mid-stance phase after SET, consistent with a small effect size (20–45% of stance, Cohen’s d = 0.20–0.42, Figure 4D).

The variance in hip flexor force was significantly reduced after SET from 20 to 75% of the stance phase, and the variance in hip flexor power was reduced from 20 to 50% and from 85 to 95% of the stance phase (*p* < 0.05; Figure 4B,D).

### 3.9. Metabolic Rate

**Figure 5:**
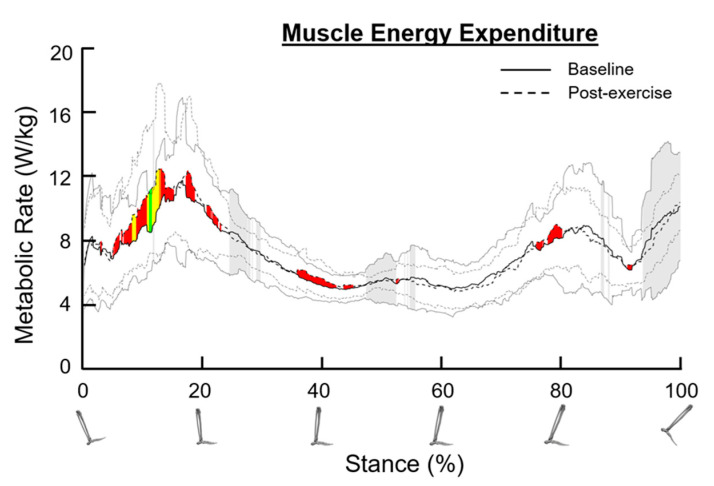
Total metabolic rate across stance for patients with peripheral artery disease. The dark solid line and the dark dotted line represent the average metabolic rate at the baseline and post-exercise sessions, respectively. The grey solid lines and the grey dotted lines represent one standard deviation from the average values. Shaded red, shaded yellow, and shaded green regions indicate small, medium, and large size effects, respectively, determined by Cohen’s d values. Shaded grey regions between standard deviation lines indicate a significant difference in variance between the baseline and post-exercise sessions (*p* < 0.05).

The mean metabolic rate significantly increased during the stance phase after SET, consistent with small, medium, and large effect sizes (early stance: 5–25%, Cohen’s d = 0.20–0.82); mid stance: 35–45%, Cohen’s d = 0.20–0.47; late stance: 75–80%, Cohen’s d = 0.20–0.36; Figure 5). Significant reductions in metabolic rate variance were also observed during the stance phase (*p* < 0.05, Figure 5).

## 4. Discussion

SET is a primary treatment for PAD [16,17,18,19]. Although SET improves quality of life and walking performance in patients with PAD [20,21,22,23,24,25], it is not well-understood how muscle function contributes to an improved walking performance after SET. To the best of our knowledge, this study is the first to utilize musculoskeletal modeling and simulation to comprehensively analyze how muscle force and power change in patients with PAD after 6 months of a SET program.

We hypothesized that muscle function would improve following SET, and our hypothesis was partially supported. More specifically, our results demonstrated an improved knee muscle function in patients with PAD during weight acceptance. Patients with PAD experienced an increase in knee extensor force during weight acceptance. However, this increase in knee extensor force did not translate to increased negative power during weight acceptance. The negative power region during weight acceptance on the knee muscle power graphs from our study aligns with the region around the peak point knee power absorption in the early stance phase [7]. Knee muscle power during weight acceptance has been shown to be attenuated in patients with PAD [7,35,36,37], and our results suggest that 6 months of SET did not lead to significant improvements in power absorption during weight acceptance, despite an increase in knee extensor force in the early stance phase.

Hip muscle forces and powers were also changed after SET in patients with PAD. During weight acceptance, the increase in hip adductor force was consistent with small and medium effect sizes, and peak hip extensor and hip flexor forces experienced a small increase. Hip abductor and hip external rotator forces remained unaltered, whereas all hip muscle groups experienced increased power during stance. Increased force in this study did not coincide with significantly increased power. Since some regions of the stance phase that had increased force did not demonstrate elevated power, the improvements in muscle power are likely associated with alterations in force and movement velocity during different stages of stance. Our results indicate that after SET, there are increases in hip forces and power that we interpret as beneficial adaptations to SET, since hip force and power are typically attenuated in PAD [7,26,35,36].

Our results indicate that positive power was increased in knee and ankle muscles following SET. In patients with PAD, it has been documented that the K2 region, which is associated with positive power production after weight acceptance, is depressed compared to controls [36,37]. Our knee muscle power graphs suggest that knee power was slightly increased in the K2 region after SET. Collectively, the plantar flexor muscles did not experience an improvement in force production despite improvements in the lateral and medial gastrocnemius muscles; however, positive power production near toe-off was improved, and this was attributed to changes in soleus, medial gastrocnemius, and lateral gastrocnemius activity. Additionally, our results indicate that knee flexor force and power production were improved in the late stance phase, near toe-off, which compliments the alterations to plantar flexor function; however, the alterations in knee flexor function should not be taken in isolation of the plantar flexors, since the gastrocnemius muscles are biarticular at the knee. The previous literature has demonstrated that plantar flexor function in PAD is attenuated during propulsion [26,35,36,37], and our results indicate that 6 months of SET could have a small impact on plantar flexor propulsion. These results suggest that there was an observable augmentation of knee flexor and plantar flexor force and power following SET, and this may translate into improvements in walking ability.

The metabolic rate was elevated during weight acceptance, single-leg support, and propulsion phases in patients with PAD after six months of SET. The metabolic rate across stance was derived using Umberger-style metabolic probes in OpenSim [31]. The increased muscle forces and powers contribute to an increased metabolic rate. During weight acceptance, the metabolic rate was elevated, aligning with the increased knee extensor force. Centralized at 40% of the stance phase, the increase in metabolic rate demonstrated a small effect size, which aligns with the increased power produced by the knee extensors in the K2 region (associated with power generation after weight acceptance). Finally, at 80% of the stance phase, the increase in metabolic rate after SET demonstrated a small effect size, and this aligns with the region where the plantar flexors and knee flexors are producing substantial power and force for propulsion at toe-off. Our metabolic probe results indicate that the early stance, mid-stance, and late stance phases all increased in metabolic rate, and that increase aligned with the elevated force and power production from multiple muscle groups. We interpret these increases in metabolic rate as being beneficial because they are coupled with increases in muscle power and force.

Previous studies have reported that patients with PAD have an altered myofiber morphology, related myopathy in the calf muscles, and reduced muscle forces and strength surrounding the ankle, knee, and hip joints [10,26,38,39]. Patients with PAD had a significantly increased proportion of gastrocnemius target myofibers and reinnervation of myofibers, with an increased myosin heavy-chain I content following SET, compared to the control group [40]. It was also reported that patients with PAD who participated in a 3-month supervised treadmill exercise had an improvement in gastrocnemius muscle microvascular flow, oxygen extraction, and oxidative metabolic capacity compared to patients with PAD who did not participate in the supervised treadmill exercise program [41]. It is believed that exercise therapy induces an adaptive response, including denervation and innervation of the gastrocnemius muscle in patients with PAD [42]. This eventually may lead to an improved walking performance in patients with PAD. Ankle plantar flexor muscle strength also significantly increased following SET [20]. Our current results utilizing modeling and simulation show that patients with PAD can generate increased force and power for some of the lower extremity muscles during walking following the SET program, which may explain how the claudication distances significantly increased in patients with PAD following SET [20].

While changes in peak values may provide some insight about how SET impacts muscle functions, variance provides additional information. It is interesting to note that there was generally a large change in force and power variances in several muscle groups after SET, but these changes were most prominent in the muscles of the knee and hip. In the muscles of the knee and hip, before SET, inter-subject variance was high among patients with PAD, but after SET, the variance decreased substantially in the muscles of the knee and the hip. Whether the significant reduction in variance for muscle force and muscle power is beneficial has yet to be fully elucidated. However, our previous studies have identified that patients with PAD have a significant increase in gait variability (standard deviation, coefficient of variation, Lyapunov exponent) at the ankle, knee, and hip joints compared to healthy older individuals [9,43]. Future studies should investigate whether the gait variability correlates with muscle force variance and how the changes in muscle force variance after SET impact overall walking performance in patients with PAD.

Simulation studies enable additional muscle contribution insights, but there are also limitations. One potential limitation was that the simulations for the baseline and post-exercise sessions were performed with default muscle properties provided by the OpenSim model [30]. We acknowledge that muscles may be altered in individuals with PAD after SET, but the details of muscle properties (maximum isometric force, optimal fiber length, maximum fiber shortening velocity, pennation angle at optimal fiber length, and tendon slack length) in patients with PAD are unexplored in the existing literature. A further potential limitation of this study is the small sample size. Another limitation is that there were no electromyography (EMG) data to validate the muscle activation patterns. However, the simulated muscle forces and activation timings are consistent with previously published data [44,45,46,47,48,49]. Finally, this study only included patients who had been diagnosed with Fontaine stage II PAD, limiting the outcomes, which may be different for other stages of PAD. Future studies should be conducted to generate a PAD model with appropriate muscle structural characteristics and to determine how those muscle characteristics are altered after SET.

## 5. Conclusions

Several key findings can be derived from our simulation results. Cohen’s d revealed that there were several small- to medium-sized effects related to muscle force and power change in the lower extremity muscles after SET. We demonstrated that metabolic rate increased in regions of stance associated with augmented force and power. We also suggest that inter-subject force and power variances were high in patients with PAD and that inter-subject variance was attenuated after SET. Overall, our results indicate that from a biomechanics perspective, muscle functions during walking are improved in patients with PAD after SET; however, the improvements were generally small.

## Data Availability

Data will be made available on request.
